# Devolution, National Pluralism and the Role of the UK Supreme Court

**DOI:** 10.1093/ojls/gqaf014

**Published:** 2025-05-13

**Authors:** Josep M Tirapu-Sanuy

**Keywords:** devolution, Supreme Court, Scotland, federalism, secession

## Abstract

This article concerns the role of the UK Supreme Court in the devolution settlement. It starts by describing the approach adopted by the Supreme Court in relation to devolution cases, characterised by a style of reasoning strictly tied to the literal meaning of the statutory text, and an expansive understanding of the principle of parliamentary sovereignty. The article argues that the purpose of devolution is the accommodation of national pluralism: devolution institutionalises the unique plurinational nature of the UK, accommodating the claims to self-government advanced by the UK’s minority nations. This has important implications for the Supreme Court: in deciding devolution cases, the Supreme Court can contribute positively or negatively to the achievement of this purpose. I maintain that the Court ought to reason and interpret the devolution statutes in a manner which promotes the accommodation of national pluralism, moving away from the current approach. The argument is illustrated with an analysis of the *IndyRef2* judgment, in comparison with the Canadian *Quebec Secession Reference*.

## 1. Introduction

The Supreme Court plays a crucial role in the UK’s territorial constitution. The powers of the devolved legislatures are limited by the devolution statutes, and conflict occasionally arises about how the provisions of those statutes ought to be interpreted. When disputes arise, courts—and most notably the Supreme Court—can be invited to step in and provide an authoritative resolution. In doing so, courts perform an authentic role of arbiters between the devolved and the central levels of government.^1^

In managing these disputes, the Supreme Court has contributed to the development and evolution of the devolution settlement.^2^ The provisions of the devolution statutes are often capable of being interpreted in various plausible ways; indeed, this is what leads to conflict, which results in referring disputes to courts. In choosing between one interpretation or another, the Supreme Court contributes to the definition of the content of those provisions, moulds the powers of the institutions of both levels of government and shapes the constitutional position of the devolved institutions. Thus, the Supreme Court inescapably plays an active role in developing the law of the UK’s territorial constitution. In other words, what the Supreme Court does is not only apply the rules of the game to adjudicate disputes between the UK and the devolved governments—it also contributes to the creation and definition of the rules of the game themselves.

By critically examining how the Supreme Court has dealt with devolution cases, this article proposes an approach for how the role of arbiter of the UK’s territorial constitution ought to be performed. As the article shows, the approach currently followed by the Supreme Court is characterised by a style of reasoning strictly tied to the literal meaning of the words used by the devolution statutes, avoiding open engagement with questions of constitutional principle. This is combined with a broad understanding of the principle of Westminster parliamentary sovereignty. In this article, I argue that this approach is damaging to the devolution settlement, and that the Supreme Court ought to be more willing to engage with the constitutional purpose of devolution.

The approach followed by the Supreme Court results from a failure to appreciate the constitutional significance of devolution, leading to a failure to properly identify what the purpose of the devolution statutes is. The article argues that the best approach would be for the Supreme Court to recognise that the purpose of devolution is *the accommodation of national pluralism*. Devolution creates political institutions at the sub-state level which accommodate the claims to self-government advanced by the UK’s minority nations, thereby giving institutional expression to the unique plurinational nature of the UK’s political community.

Acknowledging this account of devolution by the Supreme Court would promote a style of reasoning more sensitive to minority nation self-government and to the constitutional position of the minority nations in the UK constitution. Such a style of reasoning could encourage the other institutions of the UK, especially the UK government, to take national pluralism seriously and to play their part in its accommodation. This article tests this general argument by examining how the Supreme Court has decided an important devolution dispute: the *IndyRef2* case.^3^ The analysis of the case is illustrated by a comparison with the Canadian *Quebec Secession Reference* judgment.^4^

## 2. The Approach of the Supreme Court to Devolution

The powers of the devolved legislatures are subject to limitations established by the devolution statutes—the Scotland Act 1998, the Government of Wales Act 2006 and the Northern Ireland Act 1998. The most important limitations, present in the statutes of the three devolved nations, are of three kinds. First, the devolved legislatures cannot legislate on issues which relate to reserved matters, that is, a listed set of matters on which only the UK Parliament can legislate.^5^ Second, they cannot legislate to modify a list of provisions, mostly from Acts of the UK Parliament, known as protected enactments.^6^ These include, *inter alia*, the Human Rights Act 1998, the European Union (Withdrawal) Act 2018, the UK Internal Market Act 2020 and most provisions of the devolution statutes themselves. Third, the devolved legislatures cannot legislate incompatibly with rights of the European Convention on Human Rights (ECHR).^7^

Provisions in breach of these limitations are outside of the competence of the devolved legislature in question. Where a dispute arises as to whether a specific provision of a devolved piece of legislation is in breach of any of these limitations, the Supreme Court can be invited to step in and provide an authoritative resolution. These disputes can be referred to the Supreme Court by the law officers of both the UK and the devolved governments, as well as by private litigants.^8^ The provisions can be referred to the Court before their enactment, by challenging a Bill,^9^ as well as after their enactment, by challenging an Act of the devolved legislature.^10^ The number of disputes referred to the Supreme Court was low during the first nearly two decades of devolution.^11^ However, the management of Brexit, marked by ‘hyper-litigation’, has resulted in a substantial increase in the number of devolution cases reaching the Supreme Court.^12^

In dealing with these disputes, the approach followed by the Supreme Court has been characterised by a style of reasoning strictly tied to the literal meaning of the words used in the devolution statutes.^13^ In the early days of devolution, the House of Lords seemed to be more willing to openly engage with questions of constitutional principle in interpreting the devolution statutes. In *Robinson*, Lord Bingham described the Northern Ireland Act 1998 as ‘in effect a constitution’ and maintained that the provisions of that Act should ‘be interpreted generously and purposively, bearing in mind the values which the constitutional provisions are intended to embody’.^14^ However, in subsequent cases the Supreme Court moved away from this approach towards a style of reasoning strongly tied to the statutory text.

The current approach was primarily set in *Imperial Tobacco v The Lord Advocate*, where Lord Hope stated that the rules governing the competence of the Scottish Parliament ‘must be interpreted in the same way as any other rules that are found in a UK statute’^15^ and that ‘the description of the [Scotland] Act as a constitutional statute cannot be taken, in itself, to be a guide to its interpretation’.^16^ Therefore, the devolution statutes need to be ‘construed according to the ordinary meaning of the words used’.^17^ While the ‘purpose’ of the devolution statutes is still mentioned as a factor to be taken into account by the court,^18^ strong emphasis is placed on the literal meaning of the statutory text, avoiding engagement with questions of constitutional principle. Subsequent cases have generally followed the approach set in *Imperial Tobacco*, reasoning primarily on the basis of the literal meaning of the words of the devolution statutes rather than engaging with their constitutional background.^19^ This approach to devolution can be seen as part of a more general ‘restrained’ attitude adopted by the Supreme Court in public law cases.^20^

However, a closer examination of the devolution case law, especially of the most recent cases, shows that questions of constitutional principle are not totally absent from the Supreme Court’s devolution judgments. Despite its apparent style of reasoning being strictly tied to the statutory text, the Court has chosen to give primary importance to one specific constitutional principle: Westminster parliamentary sovereignty. This principle is recognised and protected by the three devolution statutes, which contain provisions stating that devolution does not affect the power of the UK Parliament to make laws for each of the devolved nations.^21^ In the *Continuity Bill* case, the Supreme Court characterised parliamentary sovereignty as reflecting the ‘essence of devolution’,^22^ and described this principle as the ‘*unqualified* legislative power’ of the UK Parliament.^23^ By using the adjective ‘unqualified’, not present in the text of any of the devolution statutes, the Court was indeed moving beyond the ‘ordinary meaning of words’ of the devolution statutes and giving effect to background constitutional principles—in this case, the principle of parliamentary sovereignty understood in a broad manner.^24^

In the *UNCRC* case, the Supreme Court provided some guidance on what it means by ‘unqualified’ legislative power, implying that parliamentary sovereignty includes some sort of immunity by the UK Parliament against external pressure. This notion led the Court to strike down, among others, a provision of a Scottish Bill which empowered courts to issue non-binding ‘incompatibility declarators’ whenever they found that a provision of a piece of legislation—either from the UK or the Scottish Parliament—was incompatible with the requirements of two international treaties ratified by the UK—the UN Convention on the Rights of the Child and the European Charter of Local Self-Government.^25^ The struck down provision had been designed as a mechanism to facilitate the incorporation of these two international conventions into Scottish law in areas of devolved competence,^26^ similar to the role that section 4 of the Human Rights Act 1998 plays in the incorporation of the ECHR into UK law. According to the Supreme Court, giving courts the power to issue incompatibility declarators would offend parliamentary sovereignty, therefore entailing a modification of section 28(7) of the Scotland Act: an incompatibility declarator

would be a judicial finding that an Act of [the UK] Parliament is incompatible with [the convention’s] obligations. The possibility—and, a fortiori, the actuality—of such finding would plainly affect Parliament’s power to make laws for Scotland, since it would *impose pressure on Parliament to avoid the opprobrium* which such a finding would entail.^27^

The idea of an unqualified legislative power reflects an expansive understanding of the principle parliamentary sovereignty, one which goes beyond guaranteeing Westminster the ongoing capacity to legislate for the minority nations: under parliamentary sovereignty as ‘unqualified legislative power’, the minority nations cannot legislate in manners which may impose ‘pressure’ on Westminster to avoid ‘opprobrium’. The adoption of this expansive interpretation, which does not straightforwardly flow from the ‘ordinary meaning’ of the text of the devolution statutes, reflects a particular constitutional vision of devolution adopted by the Court.^28^

Interpreted in such an expansive manner, the principle of parliamentary sovereignty entails a restrictive approach to the legislative powers of the devolved legislatures. As Aileen McHarg explains, section 28(7) of the Scotland Act, which was ‘previously understood as a symbolic reassertion of Westminster’s continuing sovereignty’, is converted in the *UNCRC* judgment ‘into an active, and potentially far-reaching constraint on devolved legislative freedom’.^29^ It is still not clear how far-reaching this constraint might be, as it is still not apparent what ‘unqualified’ exactly means: what qualifications exactly should the UK Parliament be free from? In *UNCRC*, we knew that parliamentary sovereignty entails immunity from unfavourable non-binding judicial rulings even in areas of devolved competence, since these would impose pressure to avoid opprobrium. But it is unclear whether it also entails immunity from other measures of a more political nature promoted by the devolved legislatures, which would equally exert pressure on the UK Parliament to avoid opprobrium.

## 3. The Constitutional Purpose of Devolution

In the previous section, I explained that the Supreme Court’s approach to devolution can be characterised by two features: a style of reasoning strictly tied to the statutory text and a broad understanding of the principle of Westminster parliamentary sovereignty. To understand and assess this approach, we must take one step backwards and consider the place that the devolution settlement occupies in the UK constitution. In this article, I argue that the Supreme Court’s approach can be seen as resulting from a failure to properly appreciate the constitutional significance of devolution, which has led the Court to inadequately identify what the purpose of the devolution statutes is. The Supreme Court has made various statements which point in different directions as to what is the purpose of the devolution statutes, but none of these statements deal with the issue in a sufficiently satisfactory manner.

What, then, is the purpose of the devolution statutes? The obvious starting point is that the purpose of the devolution statutes is to implement devolution, that is, to give effect to a profound reform of the UK’s territorial constitution which took place simultaneously in its three minority nations. Although the asymmetries within devolution are notable,^30^ especially in Northern Ireland,^31^ the Supreme Court has tended to regard the three devolution regimes as a single body of constitutional reform for the UK, giving rise to a single body of case law.^32^ Therefore, to understand the purpose of the devolution statutes we must ascertain what it is that the three devolution regimes have in common; in other words, we must ascertain what is the purpose of devolution as a distinct constitutional settlement.

In this section, I argue that the best approach is to characterise the purpose of devolution as *the accommodation of the UK’s national pluralism*. The common logic of the three devolution regimes is that they institutionalise a salient feature of the UK’s political community: its unique plurinational nature. To develop this point, the following paragraphs examine three alternative accounts of the purpose of devolution present in the Supreme Court case law: the workability account, the democracy account and the subsidiarity account. I argue that, while these accounts point at features of the devolution settlement of great importance, by themselves they do not properly reflect the constitutional significance of devolution. A fourth account, not present in the Supreme Court’s case law, is more accurate: the national pluralism account, which sees the purpose of devolution as the accommodation of national pluralism.

Before proceeding with the development of the argument, two key concepts need to be clarified—‘purpose’ and ‘constitutional significance’. By ‘purpose’ is meant the objective that a statute seeks to achieve. Admittedly, different political actors may have different competing objectives in the process of enactment of a statute. For example, devolution statutes may be seen as a stepping stone towards independence by nationalists and as a mechanism to safeguard the union by unionists. However, the argument in this article proceeds on the assumption that the law is a system which operates under its own logic, distinct from the motives pursued by political actors. Therefore, it is possible to ascertain the purpose of a statute objectively, taking into account the place that the statute occupies in the legal system.

The idea of ‘constitutional significance’ also needs to be clarified—many scholars have affirmed that devolution possesses constitutional significance without explaining what is meant by that. For the purposes of this article, we may say that the constitutional significance of a statute is the salient element that this statute adds to the structure of government of a country. For example, we may say that the Dentists Act 1984 does not possess any constitutional significance because it adds nothing salient to the UK structure of government: after the passing of the Dentists Act 1984, the UK constitution remained essentially in the same form.^33^ Conversely, we may say that the Human Rights Act 1998 possesses constitutional significance because it adds a salient element to the UK structure of government: the system of protection of human rights of the ECHR. Therefore, identifying what is the constitutional significance of devolution entails identifying *which specific salient element* devolution adds to the UK structure of government.

Any satisfactory account of the purpose of devolution must capture its constitutional significance. Therefore, alternative accounts of the purpose of devolution can be evaluated from this perspective: it is possible to assess the extent to which they reflect the constitutional significance of devolution—in other words, the extent to which they capture the specific salient element that devolution adds to the UK structure of government. In the following paragraphs, this perspective is adopted to evaluate four alternative accounts of the purpose of devolution.

The first is the workability account. According to this account, the purpose of the devolution statutes would be to create a system for the exercise of legislative power at the devolved level which is ‘coherent, stable, and workable’. The idea was first used by the Supreme Court in *Imperial Tobacco*^34^ and has subsequently been employed in other devolution cases.^35^

The workability account points at values of great importance for devolution: interpreting the devolution statutes coherently, stably and workably provides legal certainty to all the parties affected by devolved legislation making, including both public authorities and individuals. Therefore, coherence, stability and workability are important values to be considered when interpreting the devolution statutes. However, the workability account does not in itself adequately capture the constitutional significance of devolution: it fails to explain the specific salient element that devolution has added to the UK structure of government. Lord Hope admits this in *Imperial Tobacco*, explaining that these factors are ‘common to any other statute that has been enacted by the legislature, whether at Westminster or at Holyrood’.^36^ Indeed, the purpose of the Local Government Act 2000 can also be said to be to create a system of government at the local level in England and Wales which is ‘coherent, stable, and workable’,^37^ and the purpose of the Tribunals, Courts and Enforcement Act 2007 can also be said to be to establish a unified tribunal system in the UK which is ‘coherent, stable, and workable’. But we know that the devolution statutes add something salient to the UK constitution different from what is added by local government or the tribunal system, something which is not fully captured by the values of coherence, stability and workability.

An alternative account of the purpose of devolution present in the Supreme Court case law is the democracy account. According to this account, the purpose of devolution would be to promote democratic governance in the UK by creating and empowering democratically directly elected legislatures at the sub-state level. This idea finds support primarily in the Supreme Court *AXA* judgment, with Lord Hope emphasising that ‘the Scottish Parliament takes its place under our constitutional arrangements as a self-standing democratically elected legislature’^38^ rooted in ‘the traditions of a universal democracy’^39^ and Lord Reed explaining that the Scotland Act ‘established a democratically elected legislature having the power to make laws’.^40^

Democracy is indeed a key principle of devolution. The fact that the devolved legislatures are directly elected is of great importance and distinguishes them from most other public bodies. However, this fact does not in itself fully capture the constitutional significance of devolution: the democracy account, while more ambitious than the workability account, still fails to reflect the specific salient element that devolution adds to the UK constitution. Local government authorities are also elected democratically: the Local Government Act 2000 can also rightly be said to have the purpose of advancing democratic governance in the UK.^41^ Under this view, devolution could be seen as some sort of *advanced* local government, in the sense that it further promotes democracy by adding a third layer of democratic government. But the relation between devolution and local government in terms of constitutional significance is not merely one of degree: devolution does not merely add an extra element of democratic governance to the UK constitution from that already added by local government; it does something qualitatively different to that.

A third possible account of the purpose of devolution is the subsidiarity account. In *IndyRef2*, the Supreme Court stated that the Scotland Act ‘establishes and promotes a system of devolution founded on principles of subsidiarity’,^42^ and Nick Barber suggests that subsidiarity is tacitly considered by courts when resolving devolution cases.^43^ In a broad sense, subsidiarity is the idea that decisions ought to be taken as closely as possible to the citizens affected.^44^ As a constitutional principle, subsidiarity has been developed mainly in the context of EU law as a presumption in favour of Member State action in the exercise of competencies shared between the European Union and its Member States.^45^ In 2021, subsidiarity was also introduced in the preamble of the ECHR to strengthen the role of the Member States *vis-à-vis* the Strasbourg Court.^46^ The suggestion is that the same principle underpins the devolution settlement: on this view, the devolution statutes have the main purpose of bringing decision making closer to the citizens affected.

Although subsidiarity clearly has some relevance to devolution, approaching devolution in terms of subsidiarity has at least three difficulties. The first is that subsidiarity is primarily a principle of allocation and exercise of powers, rather than one of creation of institutions. It creates a presumption in favour of allocating powers to—and exercising powers by—the level of government closest to the citizens, but it says nothing about which levels of government ought to exist. The existence of various levels of government is simply presupposed.^47^ Therefore, subsidiarity can be a useful principle by which to consider which powers ought to be allocated to the devolved legislatures and which ought to be allocated to the UK Parliament, and how these powers should be exercised, but it does not answer the question of why the devolved legislatures ought to exist in the first place.^48^

Secondly, and relatedly, subsidiarity can be applied to any level of government. It could also guide the allocation of powers to the local government, and subsidiarity does indeed guide the division of powers between the European Union and its Member States, and between the European Court of Human Rights and the Member States of the Council of Europe. Thus, subsidiarity does not reflect the constitutional significance of devolution: it does not capture what is specific about devolution in the UK constitution, distinct from local government, European Council membership or EU membership before Brexit.

A third difficulty is that subsidiarity normally approaches the allocation and exercise of powers in terms of efficiency or efficacy. It is concerned about which level of government can achieve given policy objectives more efficiently or effectively.^49^ This led Paul Marquardt to criticise that ‘the underlying logic of subsidiarity reduces the claim of rightful governance to a technocratic question of functional efficiency’.^50^ Devolution may or may not have made legislation making more efficient and effective in the UK, but in any case the values of efficiency and efficacy alone do not capture the constitutional significance of devolution: as I elaborate below, devolution was established with the purpose of accommodating the UK’s national pluralism, rather than enhancing efficiency or efficacy in policy making.

Therefore, subsidiarity might be a principle of devolution, potentially guiding the allocation and exercise of powers between the devolved and the central levels, but it is certainly not *the* principle of devolution.^51^ Aware of these difficulties, Nick Barber reformulates the principle of subsidiarity in order to give it a more central role in devolution.^52^ He converts it into a principle of institution creation, rather than merely power allocation or exercise, and linked to the value of democracy, rather than efficiency or efficacy. In Barber’s account, the principle of subsidiarity requires the creation of ‘vibrant democratic units’, that is, territorial divisions which enable the establishment of legislatures that succeed in engaging with citizens and, through that engagement, produce coherent and competent legislation.^53^ Barber argues that subsidiarity is sensitive to social solidarity, since democracy will be more likely to flourish in democratic units underpinned by social solidarity. Where a national group exists, we can expect a higher degree of social solidarity to exist; in these circumstances, subsidiarity may require that the boundaries of the democratic unit track the national group.^54^

Therefore, Barber’s principle of subsidiarity, when applied to a plurinational society, seems to lead to the same conclusion than the national pluralism account of devolution defended in this article: that sub-state legislatures need to be created in order to accommodate the minority nations’ claims to self-government. However, Barber reaches that conclusion for different reasons: he is concerned about national pluralism only instrumentally, to the extent that accommodating it helps create successful legislative bodies.^55^ The constitutional significance of devolution is not fully captured by this view. Devolution, as I show below, is premised on a genuine constitutional commitment to the accommodation of national pluralism. It aims to institutionalise national pluralism not for instrumental reasons, but because it recognises national pluralism as a feature of the UK’s political community with primary normative relevance.

In sum, the accounts of the purpose of devolution present in the Supreme Court case law (the workability, democracy and subsidiarity accounts) fail to fully capture the constitutional significance of devolution—they fail to grasp the specific salient element that devolution adds to the UK structure of government. The constitutional significance of devolution is only adequately captured by a fourth alternative account, one which the Supreme Court has not embraced: the national pluralism account. According to this view, more plausible than the alternatives, the purpose of devolution is to accommodate national pluralism in the UK and to institutionalise the UK’s unique plurinational nature. Devolution statutes give institutional expression to a salient feature of the UK’s political community, namely that it is composed of various nations, each of them with their own particularities.

Nations are territorially based groups defined by objective elements—cultural features and historical circumstances—as well as by subjective elements—the prevalence of a distinct identity among its members.^56^ Nations are socially constructed,^57^ but they are nonetheless an important part of the political and social life of most modern societies.^58^ In a single state, there may be more than one nation. The UK is composed of four national communities, three of which are in a minority position—Scotland, Wales and Northern Ireland. Scotland and Wales are discrete minority nations, albeit with different histories and different levels of pre-devolution institutional autonomy. The situation of Northern Ireland is more complex: it is internally divided between two national identities, Irish and British, on non-territorial lines. Additionally, the Irish identity, unlike the Scottish and Welsh identities, extends beyond the territory of the UK state into the Republic of Ireland—which is why some people dispute that Northern Ireland can be described as one of the UK’s four nations *per se*.^59^ For the purposes of this article, I use the term ‘minority nation’ to refer to Northern Ireland as well as to Scotland and Wales, whilst bearing in mind the distinctive reality of Northern Ireland.

The devolution statutes were not enacted in a vacuum: the newly created legislatures were established on top of the pre-existing institutional and legal arrangements present in each of the three minority nations. These pre-existing arrangements were reflective of the fact that the UK was historically formed as a union between distinct nations, and that the creation of the UK never entailed the eradication of its constituent nations.^60^ Accordingly, the devolution statutes ought to be comprehended in the historical context of the UK as a union state.^61^ As James Mitchell explains, to understand devolution we must understand the different legacies of the various unions between the UK’s nations. Indeed, before the enactment of the devolution statutes, Scotland already had its separate court system, different schemes of administrative devolution had been put in place for the three minority nations and various home rule arrangements had been established for (Northern) Ireland.^62^ This historical background also explains the identities of the UK’s citizens, resulting in a state where a plurality of national identities coexist.^63^

As mentioned, devolution is premised on a constitutional commitment to the accommodation of national pluralism. It assumes that national membership has normative value:^64^ at an individual level, nations may be valued as groups the membership of which is of great importance to one’s self-identity and well-being;^65^ at a collective level, national identities can be seen as supporting bonds of solidarity necessary for the implementation of redistributive social policies and for the effective operation of deliberative practices in democratic institutions.^66^ Recognising the normative value of national identities has implications for constitutional law: where national identities are plural within a state, constitutions ought to give institutional accommodation to this pluralism.^67^ This is what devolution purports to do in the UK.

Devolution creates political institutions at the sub-state level which accommodate the claims to self-government advanced by the UK’s minority nations. Additionally, devolution introduces power-sharing arrangements in Northern Ireland to manage its internal divisions. The self-government of the minority nations is not adequately accommodated by the UK Parliament because of the way plurinationality is demographically structured in the UK: one nation, England, holds almost 85% of the population of the state, with none of the minority nations holding more than 10% of the population alone.^68^ This structurally minoritarian position leaves the minority nations in a situation of democratic vulnerability: the Scottish, Welsh and Northern Irish MPs elected to Westminster can always be outvoted by the English MPs. Devolution protects the minority nations from this democratic vulnerability by creating and empowering their own institutions of self-government, giving institutional expression to the preferences of the minority nations’ electorates.

Admittedly, concerns about democratic vulnerability could also be employed in relation to other territories of the UK, not only Scotland, Wales and Northern Ireland. The MPs elected in Cambridgeshire may also be outvoted by the MPs elected in the rest of the UK, but this does not seem to justify the creation of a parliament of Cambridgeshire. What distinguishes Cambridgeshire from the devolved nations? This is what the democracy account alone fails to explain: if the purpose of devolution had been merely to promote democratic governance, there would be no reason why the relevant constituency should not have been Cambridgeshire instead of Scotland, Wales or Northern Ireland. Indeed, under the democracy account, a more rational criterion could have been used: the newly created legislatures could have included portions of the UK’s territory with a similar population, or perhaps of a similar geographic size. Such criteria would have led to a different drawing up of the devolution boundaries.

The national pluralism account helps explain why the constitutionally relevant territories are Scotland, Wales and Northern Ireland: it is because, considering the history, the culture and the national identities of the citizens, these territories can be seen as constituting distinct national communities. The fact that the devolution boundaries were drawn on the basis of the minority nations’ boundaries reflects the purpose of accommodating and giving institutional expression to national pluralism. In this manner, the national pluralism account rightly reflects the constitutional significance of devolution—it reflects the novel salient element that devolution adds to the UK structure of government, distinct to what local government already added: after devolution, the UK constitution was transformed not merely because it became more democratic, but because it institutionalised the UK’s national pluralism. After the enactment of the devolution statutes, the UK constitution became more reflective of the plurinational nature of the political community underpinning it.

Once the national pluralism account is acknowledged, it becomes evident that the workability account and the democracy account of devolution provide only partial explanations of what devolution does. With regard to the workability account, the Supreme Court is indeed not wrong in stating that the devolution statutes intend to create a framework of legislative decision making which is ‘coherent, stable, and workable’. But the devolution statutes do that for a reason: to ensure that minority nation self-government is exercised in an effective manner. The promotion of coherence, stability and workability is instrumental to the ultimate purpose of devolution—to accommodate the UK’s national pluralism. Similarly, with regard to the democracy account, the Supreme Court in *AXA* is not wrong in pointing out the significance of the fact that the devolution legislatures are democratically elected. However, this is again instrumental to the purpose of accommodating national pluralism: the election by direct suffrage of the devolved legislatures is a marker of their constitutional status, a marker of the constitutional importance that the UK constitution gives to minority nation self-government, in contrast with previous schemes of administrative devolution. In sum, both the workability and democracy accounts provide only partial explanations, pointing at features of the devolution scheme that are in fact instrumental to the achievement of what is truly the purpose of devolution: the accommodation of national pluralism.

In the context of this discussion, one might question how effective devolution has been in accommodating national pluralism. Indeed, the profound constitutional transformation that devolution represented at the periphery was not matched by any corresponding significant changes at the centre, which remains essentially unreflective of the UK’s national pluralism.^69^ Relatedly, devolution has arguably not provided the minority nations with effective shared-rule mechanisms by which to influence decision making in London.^70^ This reflects a more fundamental concern: the lack of legal limits on the powers of the UK Parliament, reinforced by a broad understanding of the principle of parliamentary sovereignty adopted by the Supreme Court, as explained above. The only significant constraint on the powers of Westminster comes in the form of a non-enforceable political convention, the Sewel Convention, which establishes that the UK Parliament will not normally legislate with regard to devolved matters without the consent of the devolved legislatures.^71^ While the Sewel Convention was given statutory footing in the Scotland Act and the Wales Act,^72^ the Supreme Court has emphasised that it is no more than a convention; therefore, its operation and scope cannot be controlled by judges.^73^

Without tempering Westminster parliamentary sovereignty, devolution arguably offers limited protection to the minority nations: the UK Parliament has the legal capacity to overrule the democratic majorities in Scotland, Wales and Northern Ireland at any time. This has been particularly evident in the management of Brexit, which has exposed the democratic vulnerability of the minority nations.^74^ In this context, one might wonder whether national pluralism would be better accommodated through alternative constitutional arrangements, such as federalism or secession.

These deficiencies may be seen as weighing against the national pluralism account of devolution: with these limitations, it might be argued that the devolution settlement cannot be regarded as a proper solution for the accommodation of national pluralism. However, the fact that the devolution settlement does not do enough to accommodate the UK’s national pluralism does not take away that the purpose of devolution is to accommodate national pluralism. An analogy with the Human Rights Act 1998 might be illustrative at this point. It is recognised that the Human Rights Act 1998 has the purpose of giving effect to the European Convention on Human Rights in UK law, and that its constitutional significance is that it institutionalises human rights protection in the UK constitution. However, it might be argued that the Human Rights Act is an insufficient safeguard for human rights, since it leaves parliamentary sovereignty unaffected. If the UK Parliament passes an Act which blatantly violates human rights, the maximum that courts will be able to do under the Human Rights Act is to issue a non-binding declaration of incompatibility.^75^

Whether these concerns about the Human Rights Act are more or less justified, they do not affect the conclusion that the purpose of the Act is to protect human rights. These concerns merely reflect the fact that the purpose of protecting human rights had to be balanced or made compatible with the principle of parliamentary sovereignty, which is a principle of the highest importance in the UK constitution. The same is true of the devolution statutes: while one might find concerns about the perils of parliamentary sovereignty for the minority nations convincing, these concerns do not affect the conclusion that the purpose of devolution was to accommodate the UK’s national pluralism. They merely reflect the fact that this purpose was balanced or made compatible with the principle of parliamentary sovereignty.

The corollary of this discussion is that accepting the national pluralism account of devolution does not necessarily entail embracing any specific view as to the location of sovereignty in the UK’s territorial constitution. The question of sovereignty in the UK is indeed a contested matter, with competing views as to whether devolution has affected the principle of parliamentary sovereignty.^76^ However, disagreement about the location of sovereignty does not necessarily entail disagreement about the purpose of devolution. The proposition that the purpose of devolution is to accommodate national pluralism is capable of being accepted by both sides of the sovereignty debate: by those maintaining that sovereignty in the UK is shared by the various constituent nations as well as by those defending the most orthodox versions of the doctrine of Westminster parliamentary sovereignty. Indeed, the national pluralism account can be presented as the will of the UK Parliament: by enacting the devolution statutes, the UK Parliament intended to create a scheme with the purpose of accommodating national pluralism in the UK.

## 4. Implications for the Supreme Court

The previous section provided reasons in favour of the national pluralism account of devolution, arguing that devolution is better understood by acknowledging that its purpose is to accommodate the UK’s national pluralism. In this section, I analyse the implications of this account for how the Supreme Court ought to interpret the devolution statutes.

The starting point is that, by adopting the national pluralism account, the Supreme Court will be able to interpret the devolution statutes in a manner which more accurately reflects the nature of devolution as a constitutional settlement. Thus, taking into account the purpose of devolution will result in better interpretations of the devolution statutes. Devolution-related disputes arise when there are various competing plausible interpretations of a certain provision of the devolution statutes. In choosing between one interpretation and another, the accommodation of national pluralism could guide the Court’s decision. Specifically, this account would push the Court towards those interpretive choices that better promote the accommodation of national pluralism—those interpretations that are more respectful of the constitutional position of the minority nations and the legislative freedom of the devolved legislatures. In addition, in the specific context of Northern Ireland, the accommodation of national pluralism might require choosing those interpretations that are more consistent with the operation of power sharing.^77^

In the above-explained *UNCRC* case, for example, two competing interpretations of section 28(7) of the Scotland Act 1998 were in conflict: one side maintained that section 28(7) was a symbolic reassertion of Westminster parliamentary sovereignty,^78^ signalling the ongoing capacity of the UK Parliament to legislate for Scotland—but nothing beyond that. The rival interpretation of section 28(7), eventually embraced by the Court, saw this provision as entailing a more far-reaching limitation to devolved competence, protecting not only Westminster’s capacity to legislate for Scotland, but also its capacity to legislate free of some forms of external pressure (a guarantee for Westminster against ‘opprobrium’, in the words used by the Court).^79^ Faced with two potentially plausible interpretations of section 28(7), the national pluralism account would have inclined the Court towards the first interpretation, which would have been more generous with devolved legislative freedom. The rival interpretation, entailing a more far-reaching limitation, is arguably damaging to the accommodation of national pluralism. As instruments of minority nation self-government, the devolved legislatures should be able to legislate in ways which may impose political pressure on Westminster. An overly restrictive approach to devolved competence could be reinforcing the democratic vulnerability faced by the minority nations which devolution seeks to overcome.

At a more fundamental level, the national pluralism account draws attention to the crucial role that the Supreme Court inevitably plays in the achievement of (or failure to achieve) the purpose of devolution. By interpreting the devolution statutes in one way or another, the decisions of the Supreme Court affect the extent to which national pluralism is accommodated in the UK constitution. Judicial decisions, especially those of the Supreme Court, have an inescapable impact on the capacity of the constitutional system to accommodate national pluralism. From this perspective, courts are better understood not as mere passive enforcers of the devolution statutes, but as active players of the devolution settlement. By acquiring awareness of the role that they play, courts can adopt styles of reasoning more sensitive to the reality of national pluralism and to the constitutional position of the minority nations. This may often require going beyond the statutory text and articulating constitutional principles, as illustrated by the *IndyRef2* example, further expanded on in section 5 below.

The style of reasoning adopted by courts has a potential impact on the accommodation of national pluralism. It is in this context that embracing the national pluralism account by the Supreme Court would be beneficial for devolution. By engaging with the constitutional background of the devolution statutes, the Supreme Court could, when necessary, recognise the situation of democratic vulnerability faced by the minority nations and acknowledge the importance of minority nation self-government in addressing this vulnerability. This style of reasoning would in itself contribute positively to the accommodation of national pluralism, as it would entail a symbolic recognition by the highest court of the land of the constitutional position of minority nations.

Of course, accommodating national pluralism is not the sole responsibility of courts. Political institutions also have a major role to play in achieving this purpose. Constructive negotiations between governments and between political parties are essential to advancing the accommodation of national pluralism. Political accommodation has indeed been central in the operation of the devolution settlement in the UK, especially before Brexit. Instances of successful political accommodation were the Edinburgh Agreement, by which the Scottish Parliament was transferred the competence to legislate on the holding of an independence referendum in 2014,^80^ and the numerous legislative consent motions passed by the devolved legislatures to politically enable the UK Parliament to legislate in areas of devolved competence.^81^ However, the reality shows that political accommodation is not always possible and that disputes are occasionally referred to courts. While the judicialisation of disputes can be seen as a failure of politics, it can also be seen as an opportunity for courts to contribute positively to the operation of the devolution settlement.

Reasoning on the basis of the national pluralism account by courts could encourage the other institutions of the UK, especially the UK government, to take national pluralism more seriously and to play their part in the accommodation of the minority nations. In this way, judicial accommodation could foster political accommodation: the reasoning of the Supreme Court could promote the development of a constitutional environment more conducive to the accommodation of national pluralism by political institutions. Indeed, the institutions of the centre would give more serious consideration to the position of the minority nations if they knew that there is a Supreme Court in the background prepared to take the accommodation of national pluralism seriously.^82^ By promoting this constitutional environment, disagreements could be more easily worked out in the political process, making future judicial intervention less necessary. The contrast between the *IndyRef2* judgment and the Canadian *Quebec Secession Reference*, explained in section 5 below, provides a clear illustration of this point.

The approach proposed in this article might be objected to on the grounds that it promotes an overly activist attitude on the part of courts. It might be argued that the accommodation of national pluralism is an essentially political endeavour, and that recognising a role for unelected courts in this area could undermine democracy. To respond to this criticism, it should first be noted that the approach proposed in this article does not require courts to circumvent the text of the devolution statutes; quite the contrary: the national pluralism account pushes courts towards interpreting it in the manner most appropriate for the achievement of the purpose that these statutes seek to achieve. From this perspective, the recognition of a judicial role in this area can be seen as upholding the will of the UK Parliament.

However, one could still object that the inference of the purpose of the devolution statutes by courts is an unwarranted assertion of judicial power. On this view, it is Parliament, as the democratically elected branch of the constitution, who should make clear whether the devolution statutes have the purpose of accommodating national pluralism. If the purpose of a statute is not explicit in the statute, it is not for unelected courts to infer it. This is part of a more general criticism of purposive interpretations posed by advocates of textualist^83^ or intentionalist^84^ approaches.

Two things can be said in response to this objection. First, resorting to the purpose of devolution does not open the doors to as much judicial discretion as might be assumed. The purpose of accommodating national pluralism operates in the context of a wider set of constitutional rules and principles affecting the devolution settlement and the UK constitution generally. These rules and principles pose limits to the discretion of courts. They include the rules in the text of the devolution statutes, which must remain the starting point for the resolution of any devolution dispute. They also include principles such as workability, stability, coherence, democracy and arguably subsidiarity. While these principles do not provide an account of the purpose of devolution which fully captures its constitutional significance, as explained above, they are nevertheless important principles which should guide the judicial interpretation of the devolution statutes.^85^ Another principle which ought to be taken into account by courts is, of course, Westminster parliamentary sovereignty. As mentioned, the devolution statutes intend to balance the purpose of accommodating national pluralism with the principle of parliamentary sovereignty. This principle may entail limits on how far the Court might go in promoting the accommodation of national pluralism. Conversely, parliamentary sovereignty will need to be harmonised with the accommodation of national pluralism where possible—for instance, by moving away from notions of ‘unqualified’ legislative power as in *UNCRC*, as discussed above. In sum, all these rules and principles, among others, structure the way in which the court might play its role in the accommodation of national pluralism, limiting the scope for judicial creativity and tempering concerns about judicial activism.

A second response to the textualist or intentionalist objection is that the approach proposed in this article does not entail a transfer of power from an elected legislature to an unelected court. Rather, the relevant balance of powers here is between two democratically elected legislatures—the UK Parliament and the devolved legislatures. In devolution cases, what is at stake is not simply the application of judicially crafted standards against the powers of the executive or Parliament, as happens in most other constitutional disputes. In devolution disputes, the court is acting as an arbiter between two democratically elected institutions, rather than simply as a limit to the powers of one specific institution. In this context, concerns about the democratic legitimacy of judicial action are arguably less relevant.

## 5. An Illustration: The Powers of the Scottish Parliament to Legislate on an Independence Referendum

In the previous sections, I argued that the purpose of devolution is to accommodate national pluralism, and that recognising that purpose would have important implications for the role of courts. The argument was developed at a general level. In this section, I examine a specific judgment of the UK Supreme Court to illustrate how this general argument would operate in a specific case. The judgment taken as an illustration is the *Reference by the Lord Advocate of devolution issues under paragraph 34 of Schedule 6 to the Scotland Act 1998*—or, as it has come to be known, the *IndyRef2* case.^86^ To enrich the analysis, *IndyRef2* is compared with the Supreme Court of Canada’s *Quebec Secession Reference*,^87^ a seminal case in comparative secession law.^88^ From the perspective of national pluralism, the comparison between the two decisions is highly illustrative because these cases involve fairly similar disputes approached by courts in profoundly different manners.

*IndyRef2* concerns a matter of critical importance for Scottish self-government: the ability of the Scottish Parliament to legislate for the holding of an advisory referendum on independence. It is, indeed, a conflict where the different positions respond to different existing constitutional narratives as to the nature of the UK as a state^89^—at stake was nothing less than the question of whether the UK is a union based on the consent of its constituent nations. In this regard, it is a case touching upon the core of the claim to accommodation advanced by Scotland as a minority nation.

Requests for an Order in Council under section 30 of the Scotland Act to allow the Scottish Parliament to legislate for the holding of an independence referendum, as was done in 2014, were rejected by the UK government.^90^ In this context, the Scottish government drafted a Scottish Independence Referendum Bill providing for the holding of an advisory independence referendum, with the intention that the Scottish Parliament would legislate on the matter without the authorisation of the UK Parliament. Under section 31 of the Scotland Act, any Bill must be accompanied by a statement by the person introducing the Bill that, in their view, the Bill is within the competence of the Scottish Parliament. According to the Scottish Ministerial Code, this statement must be cleared with the Lord Advocate.^91^ In this case, the Lord Advocate had doubts as to whether the Scottish Independence Referendum Bill was within competence, so she referred the issue to the Supreme Court. The reference was made under paragraph 34 of Schedule 6 of the Scotland Act, which enables the law officers to refer ‘devolution issues’ to the Supreme Court.

The question posed to the Supreme Court was whether, under the Scotland Act, the Scottish Parliament has the power to legislate for the holding of an advisory referendum on Scottish independence.^92^ The Scotland Act provides that an Act of the Scottish Parliament is not law if it is outside the legislative competence of the Parliament,^93^ and that a provision is outside that competence if it relates to reserved matters.^94^ Schedule 5 of the Act lists the reserved matters, including ‘the Union of the Kingdoms of Scotland and England’^95^ and ‘the Parliament of the United Kingdom’.^96^ Thus, the specific legal question for the Supreme Court was whether a Bill providing for the holding of an advisory independence referendum relates to either the Union or the UK Parliament, which would result in it being outside of the competence of the Scottish Parliament.

The case that the proposed Bill was within competence was put forth by the Lord Advocate. Her reasoning relied on the fact that the referendum would be advisory, and therefore would have no legally binding consequences. The purpose of the Bill was not to break the Union, but merely to ascertain the views of the people of Scotland.^97^ Therefore, the holding of such a referendum would not take the question of the Union out of the UK Parliament’s hands, which would retain the final say on the matter.^98^ This reasoning was premised on a narrow interpretation of the phrase ‘relates to’ as requiring a ‘close connection’.^99^ More generally, the Lord Advocate argued that the Scottish Parliament must, as a matter of principle, have the power to ascertain the views of the electorate on particular issues by means of a referendum in order to subsequently engage in negotiations with the UK government.^100^ Further arguments were advanced by the Scottish National Party acting as intervener, relying on the right to self-determination under international law.^101^

The Supreme Court resolved that the Scottish Parliament does *not* have the power to legislate for the holding of such a referendum. In the Court’s view, the fact that the referendum was advisory did not change the conclusion: the Court had to consider the provision’s ‘effect in all the circumstances’, looking beyond purely legal effects.^102^ The referendum, while not legally binding, would be ‘an important political event’.^103^ Consequently, ‘in a constitution and political culture founded upon democracy’,^104^ a clear outcome would

strengthen or weaken the democratic legitimacy of the Union, depending on which view prevailed, and support or undermine the democratic credentials of the independence movement. It would consequently have important political consequences relating to the Union and the United Kingdom Parliament.^105^

Therefore, legislating on an advisory referendum would relate to the reserved matters of the Union and the UK Parliament; thus, it would be outside of the legislative competence of the Scottish Parliament.^106^ The arguments on the right to self-determination were dismissed expeditiously, maintaining that this right is reserved to situations of colonialism or foreign occupation;^107^ consequently, the Court stated that ‘the principle of self-determination is simply not in play here’.^108^

Had the Supreme Court followed the approach proposed in this article, embracing the national pluralism account of devolution, how would *IndyRef2* have been approached? It is at this point that the Supreme Court of Canada’s *Quebec Secession Reference* becomes relevant. The *Quebec Secession Reference* is cited by the UK Supreme Court in *IndyRef2*, but only to reject the argument on self-determination put forth by the Scottish National Party.^109^ As advanced, the comparison between the two decisions is highly illustrative because they represent styles of reasoning which are profoundly different.

Admittedly, the *Quebec Secession Reference* was not about the competence to hold a secession referendum, as was *IndyRef2*. The competence of Quebec’s institutions to call for such a referendum was taken for granted: two referendums on Quebec sovereignty, in 1980 and in 1995, had already been unilaterally called by the provincial institutions. The question was another one: whether, under the Canadian Constitution, a positive outcome on a secession referendum would confer on Quebec’s institutions a right to effect the secession of Quebec from Canada unilaterally.^110^ The Supreme Court of Canada answered that Quebec did *not* have that right.^111^ In this regard, the Supreme Court of Canada ruled in the same direction as the UK Supreme Court in *IndyRef2*, that is, against the claim advanced by the institutions of the minority nation: the outcomes of both cases rejected the arguments of the Quebec and the Scottish governments, respectively.^112^

However, the Canadian Supreme Court went beyond that, arguing that a clear result in favour of secession in a referendum would give rise to a constitutional duty to negotiate by the federal government and the other provinces with Quebec’s institutions. Specifically, the Court stated that ‘the clear expression of the desire to pursue secession by the population of a province would give rise to a reciprocal obligation on all parties to Confederation to negotiate constitutional changes to respond to that desire’.^113^ The duty to negotiate was inferred from the complex interplay of four principles which, in the Court’s view, underpin the Canadian Constitution: federalism; democracy; constitutionalism and the rule of law; and respect for minorities. According to the Court, these principles are the ‘lifeblood’^114^ of the Constitution as they ‘inform and sustain the constitutional text: they are the vital unstated assumptions upon which the text is based’.^115^

From one perspective, the *Quebec Secession Reference* can be seen as supporting the conclusion of the UK Supreme Court in *IndyRef2* that an independence referendum would carry great constitutional significance, to the extent of entailing the existence of a duty to negotiate on central state institutions.^116^ Yet, from a more elemental perspective, the *Quebec Secession Reference* represents a fundamentally different response to a constitutional conflict of a similar kind. The Supreme Court of Canada, unlike the UK Supreme Court, adopted a style of reasoning explicitly sensitive to the underlying pluralism of the Canadian society, delivering a judgment which was celebrated as a victory by both sides of the conflict.^117^

In *IndyRef2*, the UK Supreme Court followed its traditional style of reasoning used in previous devolution cases, explained above, strictly tied to the literal meaning of the words used in the devolution statutes. In this regard, the decision was unsurprising.^118^ The judgment rested on an analysis of the specific legal issue of what it means to relate to the relevant reserved matters, hardly engaging with the broader constitutional context. While the court was prepared to infer the purpose and the effect of the provisions of the specific Bill under challenge, it did not make any reference to the constitutional purpose of the Scotland Act or the devolution settlement. The profound constitutional significance of the issues at stake was reduced to a narrow question of statutory interpretation.

In contrast, in the *Quebec Secession Reference*, the Supreme Court of Canada showed an open willingness to engage with questions of constitutional background in order to resolve the complex and politically sensitive question that they had on the table, engaging with the principles underpinning the Canadian Constitution. An acknowledgement by the UK Supreme Court that the purpose of devolution is to accommodate national pluralism could have led it to adopt a style of reasoning in *IndyRef2* more similar to the one of the *Quebec Secession Reference*.^119^ The identification of the four underpinning principles of the Canadian Constitution is an interpretive exercise of a similar nature to the identification of the purpose of devolution by the UK Supreme Court that this article proposes.

By acknowledging the national pluralism account and the constitutional importance of minority nation self-government, the UK Supreme Court could have affirmed the legitimacy of the demand backed by a majority of the Scottish Parliament to hold a second independence referendum. The Canadian Supreme Court made several statements affirming the legitimacy that a hypothetical positive outcome on a secession referendum would have,^120^ maintaining that ‘the continued existence and operation of the Canadian constitutional order could not be indifferent’ to it.^121^ These affirmations followed from the recognition that the Canadian federal system ‘was a legal response to the underlying political and cultural realities that existed at Confederation and continue to exist’,^122^ and that the system ‘facilitates the pursuit of collective goals by cultural and linguistic minorities which form a majority within a particular province’.^123^ The Court recognised that the accommodation of Quebec, ‘where a majority of the population is French-speaking, and which possesses a distinct culture’, was one of the main purposes of the federal structure of government: ‘The social and demographic reality of Quebec explains the existence of a province of Quebec as a political unit and indeed, was one of the essential reasons for establishing a federal structure for the Canadian union in 1867.’^124^

What the Supreme Court of Canada was doing is to recognise that the purpose (or one of the purposes) of the Canadian territorial constitution is to accommodate Quebec. In *IndyRef2*, the UK Supreme Court could have likewise recognised that the accommodation of Scotland as a minority nation, and the accommodation of the UK’s national pluralism generally, is the purpose of the devolution settlement. This could have led the Court to recognise the constitutional significance of minority nation self-government in the UK and the relevance of a democratic demand advanced by the Scottish Parliament on an issue of such salience for national pluralism as the holding of an independence referendum. Elisenda Casanas-Adam explains that the Supreme Court in *IndyRef2* acknowledged the constitutional significance that the result of an advisory independence referendum would have, thereby rendering it outwith competence, but did not equally acknowledge the constitutional significance of the electoral majorities in numerous elections in Scotland with a clear commitment to a second Scottish independence referendum. As Casanas-Adam argues, ‘These are also a clear expression of the view of the Scottish electorate which, if not appropriately acknowledged and addressed, are progressively weakening the democratic legitimacy of the Union’.^125^

Just as the Canadian constitutional order ‘cannot remain indifferent’^126^ to a clear majority of Quebecers in favour of secession, so the UK constitutional order cannot remain indifferent to a clear majority in the Scottish Parliament, backed by numerous election results, in favour of holding an independence referendum, even if the Scottish Parliament does not have legal competence to legislate on that matter. The mere recognition of this fact by the Supreme Court, regardless of the outcome of the ruling, would have been beneficial to the devolution settlement: the affirmation by the highest court of the land of the legitimacy of the demand pursued by the Scottish Parliament would have represented a symbolic recognition of the constitutional importance of minority nation self-government in the UK.

Arguably, the UK Supreme Court’s approach in *IndyRef2* may even be seen as *deligitimising* the demand of the Scottish Parliament. By addressing the arguments of the Scottish National Party, the Court solemnly affirmed that ‘the principle of self-determination is simply not in play here’.^127^ To reach that conclusion, the UK Supreme Court relied on the *Quebec Secession Reference*. However, the truth is that the Canadian judgment does not contain any statement supporting this affirmation. The conclusion that can be drawn from the Canadian judgment is a narrower one: that Scotland does not have a *legal* right to *external* self-determination *under international law*. From the perspective of the accommodation of national pluralism, the affirmation that ‘self-determination is simply not in play’ is unhelpful.^128^ Self-determination may still be in play as a *political* principle invoked by the Scottish institutions in negotiating with the UK institutions,^129^ a principle which may arise from the UK constitution rather than from international law.^130^ The absolute rejection by the Supreme Court of the principle of self-determination, which was unnecessary for the Court to reach the conclusion that was intending to reach, weakens the legitimacy of the political demand advanced by Scottish institutions and runs against the accommodation of Scotland as a minority nation.

Additionally, a different style of reasoning by the UK Supreme Court could have encouraged the other constitutional actors, especially the UK government, to take national pluralism more seriously. In the *Quebec Secession Reference*, the Supreme Court of Canada adopted an explicit facilitator role, promoting that the consequences of a hypothetical secession referendum be managed in the political arena through a negotiation process.^131^ The duty to negotiate, which the Court derived from the four underpinning principles of the Canadian Constitution, can be seen as a facilitative mechanism: it forces the federal government and the other provinces to take the demands of a majority in Quebec seriously, thereby facilitating that the consequences of those demands are dealt with through a negotiated political process.

In *IndyRef2*, adopting a style of reasoning more sensitive to national pluralism could have also encouraged the UK government to take the demands of the Scottish Parliament more seriously. The point is not necessarily that the UK Supreme Court should have inferred a ‘duty to negotiate’ like the Canadian one. The UK Supreme Court could, perhaps, have signalled that by avoiding engagement with the demands of the Scottish Parliament, the UK government is hindering the accommodation of Scotland within the UK and, therefore, undermining the devolution settlement, even if the Scottish Parliament lacks legal competence to legislate on that matter. Shona Wilson Stark and Raffael Fasel make a similar point, maintaining that the Supreme Court could have stated that the UK government’s actions are ‘constitutionally improper’.^132^ According to them, this statement could have been grounded by the Supreme Court on the principle of democracy, relying on *AXA* and on the democratically elected nature of the Scottish Parliament. The suggestion here is that, further than relying on the principle of democracy, the Court could have reasoned on the basis of the importance of accommodating Scotland as a minority nation within the UK.

Would this different style of reasoning have ultimately led to a different outcome on the legal question at stake in *IndyRef2*? It would, at least, have strengthened the arguments in favour of the Bill being within competence. More specifically, the national pluralism account could have provided a basis on which to argue that Scottish independence is a matter of crucial importance for the claim of self-government advanced by Scotland as a minority nation, which devolution seeks to accommodate. Consequently, provided that there is an available plausible interpretation of the clauses of the Scotland Act, the accommodation of national pluralism requires reading the Act in a manner which allows the Scottish Parliament to ascertain the views of the Scottish people in such an important matter.^133^ Denying this capacity to the Scottish Parliament would be detrimental to the accommodation of national pluralism, thereby hindering the achievement of the purpose of the devolution settlement.

A reasoning on the basis of the national pluralism account of devolution would have pushed towards a narrower interpretation of what is meant by ‘to relate to’ a reserved matter in section 29 of the Scotland Act, giving less weight to the practical or political effects of the Bill and placing more emphasis on the bindingness of the referendum. Indeed, the emphasis on the political effects of the referendum can be seen as following a similar logic to the problematic approach adopted in the *Continuity Bill* and *UNCRC* cases in relation to parliamentary sovereignty,^134^ discussed in section 2 above.

A narrower interpretation of section 29 of the Scotland Act, leading to a different outcome, could have been backed by the acknowledgement that devolution originates from the need to overcome the democratic vulnerability of the minority nations in the UK. From this perspective, recognising the possibility to hold an advisory referendum would help protect the position of Scotland as a minority nation within the UK since it would give the Scottish institutions more capacity to exert political pressure on the UK Parliament, the only institution with legal capacity to change the law on the matter. Under this line of argument, the fact that a referendum is a particularly effective tool of political pressure would not have pushed in favour of rendering it outwith competence, but quite the contrary: due to Scotland constituting a minority within the UK, it is important to preserve the possibility for Scottish institutions to resort to the tools of political pressure that are most effective. Denying the possibility of using such a politically effective tool could leave Scotland as a minority nation vulnerable within the UK, weakening the devolution settlement.

In any case, irrespective of the outcome reached, what is clear is that adopting the national pluralism account would have led the Supreme Court to deliver a judgment that would have been much more respectful of the constitutional position of the devolved legislatures, a judgment which would have promoted rather than hindered the flourishing of the devolution settlement.

The point advanced here is not that the UK Supreme Court should have decided *IndyRef2* in exactly the same way that the Supreme Court of Canada decided the *Quebec Secession Reference*. Admittedly, the two supreme courts operate in significantly different constitutional settings. The existence of a strong principle of parliamentary sovereignty in the UK, which is not present in Canadian constitutional law, and the uncodified nature of the UK constitution may be seen as limiting the scope of action of the UK Supreme Court more than that of the Supreme Court of Canada. But despite these differences, the stark contrast between the two judicial decisions is very illustrative: it shows how the Supreme Court of Canada, faced with a profound constitutional conflict, sees itself *as playing a role* in the accommodation of Quebec and national pluralism in Canada. The result is a judgment which positively contributes to the preservation and flourishing of the Canadian territorial constitution. In contrast, the UK Supreme Court in *IndyRef2* sees itself *as playing no role at all* in this regard, refusing to acknowledge the great impact that its decisions have on the way in which the UK constitutional system accommodates Scotland as a minority nation, and national pluralism generally.

## 6. Conclusion

The current approach followed by the UK Supreme Court in relation to devolution is characterised by a style of reasoning strictly tied to the literal meaning of words, combined with a broad understanding of the principle of Westminster parliamentary sovereignty. This article has shown that this approach results from a failure by the Supreme Court to properly consider the constitutional significance of devolution. Once it is acknowledged that the purpose of devolution is to accommodate the UK’s national pluralism, the role of the Supreme Court can be better understood and evaluated. By recognising the situation of democratic vulnerability faced by the minority nations of the UK, and the importance of minority nation self-government in overcoming this vulnerability, the decisions of the Supreme Court could contribute positively to the preservation and flourishing of devolution. More fundamentally, this article has shown that the role of the Supreme Court is not one of mere passive enforcer of the devolution statutes; it inevitably plays an active role, as its decisions affect the capacity of the UK constitution to accommodate national pluralism.

## Footnotes

1 Elisenda Casanas-Adam, ‘Brexit and the Mechanisms for the Resolution of Conflicts in the Context of Devolution: Do We Need a New Model?’ in Aileen McHarg, Jo Murkens and Oran Doyle (eds), *The Brexit Challenge for Ireland and the United Kingdom: Constitutions Under Pressure* (CUP 2021).

2 Christopher McCorkindale, Aileen McHarg and Paul F Scott, ‘The Courts, Devolution, and Constitutional Review’ (2017) 36 UQLJ 289.

3
*Reference by the Lord Advocate of devolutions issues under paragraph 34 of Schedule 6 to the Scotland Act 1998* [2022] UKSC 31.

4
*Reference re Secession of Quebec* [1998] 2 SCR 217.

5 Scotland Act 1998, s 29(2)(b), with the reserved matters listed in sch 5; Government of Wales Act 2006, s 108A(2)(c), with the reserved matters listed in sch 7A; Northern Ireland Act 1998, s 6(2)(b), with the excepted matters listed in sch 2 and the reserved matters listed in sch 3.

6 Scotland Act 1998, s 29(2)(c), with the protected enactments listed in sch 4; Government of Wales Act 2006, s 108A(2)(d), with the protected enactments listed in pt 1 of sch 7B; Northern Ireland Act 1998, s 6(2)(f), with the protected enactments listed in s 7.

7 Scotland Act 1998, s 29(2)(d); Government of Wales Act 2006, s 108A(2)(e); Northern Ireland Act 1998, s 6(2)(c).

8
*AXA General Insurance Limited and others v The Lord Advocate and others* [2011] UKSC 46.

9 Scotland Act 1998, s 33; Government of Wales Act 2006, s 112; Northern Ireland Act 1998, s 11. In *IndyRef2* (n 3), it was confirmed that Bills can be referred under para 34 of sch 6 of the Scotland Act 1998 by the Lord Advocate to the Supreme Court before being introduced.

10 Scotland Act 1998, sch 6; Government of Wales Act 2006, sch 9; Northern Ireland Act 1998, sch 10.

11 Aidan O’Neill, for example, wrote in 2013 that ‘there have been surprisingly few challenges to the validity of provisions of Acts of the Scottish Parliament (despite its prodigious output of legislation since it opened for business in 1999)’. Chris McCorkindale and Janet Hiebert attributed this low amount of litigation to the successful operation of the system of parliamentary review of legislative competence established by the Scotland Act. Aidan O’Neill, ‘Back to the Future: Judges, Politicians and the Constitution in the New Scotland’ (2013) 18 JR 45, 57; Christopher McCorkindale and Janet L Hiebert, ‘Vetting Bills in the Scottish Parliament for Legislative Competence’ (2017) 21 Edinburgh Law Review 319.

12 Christopher McCorkindale and Aileen McHarg, ‘Litigating Brexit’ in McHarg, Murkens and Doyle (n 1).

13 Chris McCorkindale, ‘Devolution in Scotland and the Supreme Court: A Question of Interpretation?’ (2020) 50 Materiali per una storia della cultura giuridica 67.

14
*Robinson v Secretary of State for Northern Ireland* [2002] UKHL 32, 11.

15
*Imperial Tobacco v The Lord Advocat*e [2012] UKSC 61, 14.

16 ibid 15.

17 ibid 14.

18 ibid 15.

19 Nick Barber, *The United Kingdom Constitution: An Introduction* (OUP 2021) ch 18; Nicholas Kilford, ‘Interpreting the Devolution Statutes’ (UK Constitutional Law Association, 28 May 2024) <https://ukconstitutionallaw.org/2024/05/28/nicholas-kilford-interpreting-the-devolution-statutes/>.

20 Lewis Graham, ‘Has the UK Supreme Court Become More Restrained in Public Law Cases?’ (2024) 87 MLR 1073.

21 Scotland Act 1998, s 28(7); Government of Wales Act 2006, s 107(5); Northern Ireland Act 1998, s 5(6).

22
*The UK Withdrawal from the European Union (Legal Continuity) (Scotland) Bill—A Reference by the Attorney General and the Advocate General for Scotland* [2018] UKSC 64, 41 (*Continuity Bill*).

23 ibid 52 (emphasis added).

24 For an analysis of how the principle of parliamentary sovereignty informed the *Continuity Bill* judgment, see Aileen McHarg and Chris McCorkindale, ‘The Supreme Court and Devolution: The Scottish Continuity Bill Reference’ [2019] JR 190, especially 195–6.

25
*Reference by the Attorney General and the Advocate General for Scotland—United Nations Convention on the Rights of the Child (Incorporation) (Scotland) Bill and European Charter of Local Self-Government (Incorporation) (Scotland) Bill* [2021] UKSC 42, 47–55 (*UNCRC*).

26 The judgment recognised the possibility for the Scottish Parliament to legislate to incorporate ratified international treaties into Scottish law in areas of devolved competence. See ibid 4.

27 ibid 52 (emphasis added). One surprising element in the Court’s reasoning is the way in which incompatibility declarators under the Scottish Bills are compared with declarations of incompatibility under s 4 of the Human Rights Act 1998 (HRA). According to the Court, s 4 HRA entails a qualification of parliamentary sovereignty, but this is not problematic, since ‘[the UK] Parliament can itself qualify its own sovereignty … The Scottish Parliament, on the other hand, cannot qualify the sovereignty of [the UK] Parliament’. This seems a departure from previous understandings of s 4 HRA, which was generally regarded as fully consistent with parliamentary sovereignty, rather than as a qualification to it. For a more detailed analysis of this point, see Mark Elliott and Nicholas Kilford, ‘Devolution in the Supreme Court: Legislative Supremacy, Parliament’s “Unqualified” Power, and “Modifying” the Scotland Act’ (UK Constitutional Law Association, 15 October 2021).

28 Gregory Davies takes this interpretation as evidence that the rulings of the Supreme Court have served to centralise state authority in the UK. Interestingly, Davies adopts a Marxist perspective, arguing that the Court has been entrusted with the role of ‘guardian of the passive revolution’, responsible for containing and disciplining peripheral pressures to ensure that the concessions granted to the minority nations under devolution remain limited in nature. See Gregory Davies, ‘The UK Supreme Court and Devolution: Guardian of the Passive Revolution?’ [2025] PL 58.

29 Aileen McHarg, ‘Devolution: A View from Scotland’ (Constitutional Law Matters, 23 May 2022) <https://constitutionallawmatters.org/2022/05/23/devolution-a-view-from-scotland/>.

30 Brice Dickson, ‘Work in Progress. A Country Study of Constitutional Asymmetry in the United Kingdom’ in Patricia Popelier and Maja Sahadžić (eds), *Constitutional Asymmetry in Multinational Federalism: Managing Multinationalism in Multi-tiered Systems* (Springer International Publishing 2019); James Mitchell, *Devolution in the UK* (Manchester UP 2009).

31 Christopher McCrudden, ‘Northern Ireland and the British Constitution Since the Belfast Agreement’ in Jeffrey Jowell and Dawn Oliver (eds), *The Changing Constitution* (6th edn, OUP 2007).

32 Lord Reed, ‘Scotland’s Devolved Settlement and the Role of the Courts’, The Inaugural Dover House Lecture (2019) 18.

33 The idea of the Dentists Act follows the example used by Dicey. He argued that an implication of parliamentary sovereignty is that ‘neither the Act of Union with Scotland nor the Dentists Act, 1878, has more claim than the other to be considered a supreme law’. See Albert Venn Dicey, *Introduction to the Study of the Law of the Constitution* (Liberty Fund 1893) 78.

34
*Imperial Tobacco* (n 15) 14.

35
*Continuity Bill* (n 22) 12; *UNCRC* (n 25) 7.

36
*Imperial Tobacco* (n 15) 14.

37 The same can be said of the Local Government etc (Scotland) Act 1994 or the Local Government Act (Northern Ireland) 1972.

38
*AXA General Insurance Limited and others v The Lord Advocate and others* [2011] UKSC 46, 46.

39 ibid 49.

40 ibid 145.

41 The same can be said of the Local Government etc (Scotland) Act 1994 or the Local Government Act (Northern Ireland) 1972.

42
*IndyRef2* (n 3) 90.

43 Barber, *The United Kingdom Constitution* (n 19) 279–84.

44 Preamble of the Treaty of the European Union. Note that subsidiarity is a complex concept that has been conceptualised and theorised from different perspectives. See Andreas Føllesdal, ‘Competing Conceptions of Subsidiarity’ (2014) 55 Nomos 214.

45 Treaty of the European Union, art 5.3.

46 Reto Walther, ‘Subsidiarity in the ECHR: An Empty Promise for Local Authorities?’ (2024) 28 International Journal of Human Rights 1086.

47 The EU subsidiarity principle establishes a presumption in favour of Member States *vis-à-vis* EU institutions, presupposing the existence of the two levels. The ECHR subsidiarity principle establishes that the Member States have a primary responsibility in securing the Convention rights, and that the European Court of Human Rights only has a supervisory role, again presupposing the existence of the two levels.

48 Jo Hunt, for example, analyses the UK’s territorial constitution from the lens of subsidiarity in the context of the EU, but she focuses on the allocation and exercise of powers between the devolved level and the central level. Jo Hunt, ‘Subsidiarity, Competence, and the UK Territorial Constitution’ in McHarg, Murkens and Doyle (n 1).

49 Notably, the subsidiarity test in art 5.3 of the Treaty of the European Union asks whether given policy objectives can be sufficiently achieved by the Member States, and whether the Union can achieve these objectives better.

50 Paul Marquardt, ‘Subsidiarity and Sovereignty in the European Union’ (1994) 18 Fordham Int’l LJ 616, 618.

51 This also seems to be the position adopted by the House of Lords Constitution Committee: it regards subsidiarity as one of the principles of devolution, and it describes the principle as a useful benchmark against which to test proposals for the devolution of further powers. See ‘The Union and Devolution’ (House of Lords Select Committee on the Constitution 2016) 10, 195–8.

52 Nick Barber, *The Principles of Constitutionalism* (OUP 2018) ch 7; Barber, *The United Kingdom Constitution* (n 19) ch 18.

53 Barber, *The Principles of Constitutionalism* (n 52) 195.

54 ibid 196.

55 ibid 216. In Barber’s words, ‘Subsidiarity does not endorse the nationalist beliefs that it accommodates’.

56 Stephen Tierney, *Constitutional Law and National Pluralism* (OUP 2005) 34.

57 Benedict Anderson, *Imagined Communities: Reflections on the Origin and Spread of Nationalism* (Verso 2006).

58 Michael Billig, *Banal Nationalism* (SAGE Publications 1995).

59 Some scholars have described the societies of the kind of Northern Ireland as ‘national minorities’, instead of ‘minority nations’. Seymour, Couture and Nielsen describe a national minority as a ‘partial extension of a closely situated nation on the territory of another nation’. See Michel Seymour, Joceyline Couture and Kai Nielsen, ‘Introduction: Questioning the Ethnic/Civic Dichotomy’ in Joceyline Couture, Kai Nielsen and Michel Seymour (eds), *Rethinking Nationalism* (University of Calgary Press 1998) 46.

60 James Mitchell, *Devolution in the UK* (Manchester UP 2009) 4.

61 Michael Keating, *State and Nation in the United Kingdom: The Fractured Union* (OUP 2021).

62 For a detailed overview of these pre-existing institutional and legal arrangements and the way in which they informed devolution, see Mitchell (n 60).

63 Keating (n 61) esp ch 10.

64 On this point, the national pluralism account of devolution differs from Nick Barber’s account of the principle of subsidiarity. As seen above (n 55), Barber’s principle does not ascribe normative value to national identities. It seeks to accommodate them only for instrumental reasons.

65 Avishai Margalit and Joseph Raz, ‘National Self-Determination’ (1990) 87 Journal of Philosophy 439; Will Kymlicka, *Multicultural Citizenship: A Liberal Theory of Minority Rights* (OUP 1996) esp ch 6.

66 David Miller, *On Nationality* (OUP 1995) esp ch 4.

67 Tierney, *Constitutional Law and National Pluralism* (n 56); Will Kymlicka, ‘Federalism, Nationalism, and Multiculturalism’ in Dimitros Karmis and Wayne Norman (eds), *Theories of Federalism* (Springer 2005); Alain-G Gagnon, *The Legitimacy Clash. Challenges to Democracy in Multinational States* (University of Toronto Press 2023).

68 Office for National Statistics, ‘Population Estimates for the UK, England, Wales, Scotland, and Northern Ireland: Mid-2022’ (26 March 2024) <www.ons.gov.uk/peoplepopulationandcommunity/populationandmigration/populationestimates/bulletins/annualmidyearpopulationestimates/mid2022>.

69 Keating (n 61) ch 5.

70 Nicola McEwen, ‘The Limits of Self Rule Without Shared Rule’ in Ferran Requejo and Marc Sanjaume-Calvet (eds), *Defensive Federalism: Protecting Territorial Minorities from the ‘Tyranny of the Majority’* (Routledge 2022). However, some scholars have emphasised that certain mechanisms of shared rule do exist in the UK: for example, Tierney argues that the House of Lords Constitution Committee has adopted a role comparable to that of a federal second chamber, and Barber emphasises the role of interactions between Westminster and the devolved legislatures. See Stephen Tierney, ‘Towards a Principle of Positive Engagement?: The House of Lords Constitution Committee Reports on The Governance of the Union’ (UK Constitutional Law Association, 27 September 2024) <https://ukconstitutionallaw.org/2024/09/27/stephen-tierney-towards-a-principle-of-positive-engagement-the-house-of-lords-constitution-committee-reports-on-the-governance-of-the-union/>; Barber, *The United Kingdom Constitution* (n 19) 287–9.

71 For an analysis of the Sewel Convention and its operation, see Aileen McHarg, ‘Constitutional Change and Territorial Consent: The Miller Case and the Sewel Convention’ in Mark Elliott, Jack Williams and Alison Young (eds), *The UK Constitution After Miller: Brexit and Beyond* (Hart Publishing 2018).

72 Scotland Act 1998, s 28(8); Government of Wales Act 206, s 107(6).

73
*Miller v Secretary of State for Exiting the European Union* [2017] UKSC 5, 146.

74 Aileen McHarg, ‘Unity and Diversity in the United Kingdom’s Territorial Constitution’ in Mark Elliott, Jason NE Varuhas and Shona Wilson Stark (eds), *The Unity of Public Law? Doctrinal, Theoretical and Comparative Perspectives* (Hart Publishing 2018).

75 But note that declarations of incompatibility are highly effective, as mentioned in n 82 below.

76 Although the Supreme Court has generally embraced an orthodox view of parliamentary sovereignty in relation to devolution, as explained in s 2 above, there are dicta suggesting that devolution may have somehow transformed sovereignty. Specifically, in *Jackson v Attorney General* [2005] UKHL 56, 102, Lord Steyn stated *obiter dictum* that ‘The classic account given by Dicey of the doctrine of the supremacy or Parliament, pure and absolute as it was, can now be seen to be out of place in the modern United Kingdom’, and that ‘The settlement contained in the Scotland Act 1998 also point to a divided sovereignty’.

77 This was explicit in the Supreme Court’s reasoning in *Robinson v Secretary of State for Northern Ireland* [2002] UKHL 32.

78 McHarg, ‘Devolution’ (n 29).

79
*UNCRC* (n 25) 52.

80 Agreement between the United Kingdom Government and the Scottish Government on a referendum on independence for Scotland (Edinburgh, 15 October 2012) </https://assets.publishing.service.gov.uk/government/uploads/system/uploads/attachment_data/file/313612/scottish_referendum_agreement.pdf>.

81 According to the Institute for Government in an explainer dated in 2018, out of more than 400 legislative consent motions, on just 20 occasions has a devolved legislature denied consent, in part or in full. See Institute for Government, ‘Sewel Convention’ (16 January 2018) <www.instituteforgovernment.org.uk/explainer/sewel-convention>.

82 While the management of Brexit has exacerbated the tension between the courts and the executive, in normal times we should expect the executive to have proper regard to the pronouncements of judges, especially to those of the Supreme Court. The high effectivity of declarations of incompatibility, issued by courts under s 4 HRA, shows that the government generally respects judicial rulings. See Ministry of Justice, ‘Responding to Human Rights Judgments’ (November 2024) 44. Admittedly, there are important differences in the role of courts and the constitutional context between the HRA and devolution. But, in any case, the general willingness of the executive to comply with declarations of incompatibility suggests that the attitude of the recent Conservative governments in relation to judicial might be more of an exception in constitutional terms, rather than the new norm.

83 See eg Antonin Scalia and Bryan A Garner, *Reading Law: The Interpretation of Legal Texts* (Thomson/West 2012) 18–28.

84 See eg Jeffrey Goldsworthy, *Parliamentary Sovereignty: Contemporary Debates* (CUP 2010) 254–63.

85 See s 3 above.

86 Above n 3.

87
*Reference re Secession of Quebec* [1998] 2 SCR 217 (*Quebec Secession Reference*).

88 Giacomo Delledonne and Giuseppe Martinico (eds), *The Canadian Contribution to a Comparative Law of Secession: Legacies of the Quebec Secession Reference* (Palgrave Macmillan 2019).

89 Elisenda Casanas-Adam, ‘An Unanswered or Unanswerable Question? The Scottish Parliament’s Competence to Legislate for an Independence Referendum for Scotland’ [2017] Revista Catalana de Dret Públic 81.

90 Letter from Prime Minister Boris Johnson to Scottish First Minister Nicola Sturgeon, 14 January 2020 <www.gov.uk/government/publications/letter-from-pm-boris-johnson-to-scottish-first-minister-nicola-sturgeon-14-january-2020>. More generally, see David Torrance, ‘Scottish Independence Referendum: Legal Issues’ (House of Commons Library 2023) <https://commonslibrary.parliament.uk/research-briefings/cbp-9104/>.

91 Scottish Ministerial Code, para 3.4.

92 This was the main legal question of the case, on which this article focuses. But the Supreme Court had to resolve two other questions as well, on which this article does not focus: first, whether the referred question was a ‘devolution issue’ and, therefore, whether it could be the subject of a reference under para 34 of sch 6; and second, whether the court should decline to accept the reference in the exercise of its discretion.

93 Scotland Act 1998, s 29(1).

94 ibid 29(2).

95 ibid sch 5, para 1(b).

96 ibid sch 5, para 1(c).

97
*IndyRef2* (n 3) 66.

98 ibid 64.

99 ibid 65.

100 ibid 68. This line of argument had been developed by Nick Barber. See Nick Barber, ‘The Constitutional Regulation of Scottish Secession’ (12 June 2015).

101
*IndyRef2* (n 3) 84–5.

102 ibid 74.

103 ibid 81.

104 ibid.

105 ibid.

106 For a more extensive explanation of the judgment, see Andrew Sanger and Alison L Young, ‘An Involuntary Union? Supreme Court Rejects Scotland’s Claim for Unilateral Referendum on Independence’ (2023) 82 CLJ 1.

107
*IndyRef2* (n 3) 88–9.

108 ibid 88.

109 ibid 88. For further analysis of the UK Supreme Court’s treatment of the issue of self-determination, see below.

110 This was the question *on constitutional law* that the Supreme Court of Canada addressed, and which provides the lessons for comparison in this article. However, it should be noted that the Supreme Court of Canada also addressed the question of whether Quebec had a right to secession *under international law*.

111
*Quebec Secession Reference* (n 87) 91.

112 It should be noted that the government of Quebec did not participate in the proceedings before the Canadian Supreme Court, but its position was defended by an *amicus curiae*.

113
*Quebec Secession Reference* (n 87) 88.

114 ibid 51.

115 ibid 49.

116 Leah Trueblood, ‘The Impact of Federalism on Secession Referendums: Comparing Scotland and Québec’ (2023) 34 KLJ 321, 331.

117 Jean Leclair, ‘Constitutional Principles in the Secession Reference’ in Peter Oliver, Patrick Macklem and Nathalie Des Rosiers (eds), *The Oxford Handbook of the Canadian Constitution* (OUP 2017) 1010.

118 Matthew Psycharis and Alistair Mills, ‘The Scottish Parliament, the Supreme Court, and an Independence Referendum?’ (2023) 28 JR 43, 60.

119 As Stephen Tierney explained years before *IndyRef2* was decided, the ruling of the Supreme Court of Canada ‘may offer useful lessons’ to the UK Supreme Court ‘when faced with similar disputes which also, implicitly, call upon [it] to articulate the nature of the plurinational state’. Tierney, *Constitutional Law and National Pluralism* (n 56) 283.

120
*Quebec Secession Reference* (n 87) 66, 88.

121 ibid 151.

122 ibid 43.

123 ibid 59.

124 ibid 59. For a critical view, see Gregory Millard, ‘The Secession Reference and National Reconciliation: A Critical Note’ (1999) 14 Canadian Journal of Law and Society 1. Millard criticised the Court for not being explicit enough in recognising Canada as a ‘multination’ (or plurinational) polity.

125 Elisenda Casanas-Adam, ‘The Supreme Court’s Independence Referendum Decision: What Next for the Scottish Independence Referendum Process?’ (2023) 27 Edinburgh Law Review 213, 215.

126
*Quebec Secession Reference* (n 87) 92.

127
*IndyRef2* (n 3) 88. For other commentary on this point, see Lea Raible, ‘Self-Determination at the UK Supreme Court and the Failure of International Law’ (2023) 27 Edinburgh Law Review 219.

128 This view is shared by Michael Keating, ‘Unhelpful Clarifications’ (Centre on Constitutional Change, 24 November 2022) <www.centreonconstitutionalchange.ac.uk/news-and-opinion/unhelpful-clarifications>.

129 The view that Scotland has a political right to self-determination finds support in ‘The Claim of Right for Scotland’, a document signed by the cross-party Scottish Constitutional Convention in 1989 which was central to the establishment of the Scottish Parliament. Specifically, the Scottish Constitutional Convention acknowledged ‘the sovereign right of the Scottish people to determine the form of Government best suited to their needs’. The Smith Commission report, on which the Scotland Act 2016 was based, also recognised the same idea. See ‘Report of the Smith Commission for Further Devolution of Powers to the Scottish Parliament’ (The Smith Commission 2014) 20.

130 Additionally, the relevant question at stake arguably affected the *internal* dimension of the right to self-determination, that is, the right to self-government of Scotland within the UK, rather than its *external* dimension: the argument advanced by the Lord Advocate was not that the Scottish Parliament had a right to effect secession, but that it had a right to consult the Scottish people on that matter. It was, therefore, an argument about the scope of the self-government of the Scottish Parliament within the UK, rather than about secession itself.

131 Robert Schertzer, *The Judicial Role in a Diverse Federation: Lessons from the Supreme Court of Canada* (University of Toronto Press 2016) 160.

132 Shona Wilson Stark and Raffael N Fasel, ‘Unconstitutionally Legal: How the UK Supreme Court Should Decide the Lord Advocate’s Reference’ (UK Constitutional Law Association, 3 October 2022) <https://ukconstitutionallaw.org/2022/10/03/shona-wilson-stark-and-raffael-fasel-unconstitutionally-legal-how-the-uk-supreme-court-should-decide-the-lord-advocates-reference/>.

133 In the words of Nick Barber, ‘The purpose of the Scotland Act was, in part, to create a forum able to express the interests and views of the Scottish people; denying it the power to hold advisory referendums would restrict its ability to achieve this goal’. See Barber, ‘The Constitutional Regulation of Scottish Secession’ (n 100) 9.

134 As Matthew Psycharis and Alistair Mills explain, the reasoning in *Continuity Bill* and *UNCRC*, ‘though dealing with a different provision of the Scotland Act, appears at home with the reasoning [in IndyRef2]: namely, that laws sought to be enacted by a devolved legislature, the effects of which are purely political or “practical”, might still bear upon the legal norms of the devolution settlement in the relevant ways’. Psycharis and Mills (n 118) 47.

